# Age- and Gender-Related Changes in Contractile Properties of Non-Atrophied EDL Muscle

**DOI:** 10.1371/journal.pone.0012345

**Published:** 2010-08-23

**Authors:** Stephen Chan, Stewart I. Head

**Affiliations:** School of Medical Sciences, University of New South Wales, Sydney, New South Wales, Australia; University of Queensland, , Australia

## Abstract

**Background:**

In humans, ageing causes skeletal muscles to become atrophied, weak, and easily fatigued. In rodent studies, ageing has been associated with significant muscle atrophy and changes in the contractile properties of the muscles. However, it is not entirely clear whether these changes in contractile properties can occur before there is significant atrophy, and whether males and females are affected differently.

**Methods and Results:**

We investigated various contractile properties of whole isolated fast-twitch EDL muscles from adult (2–6 months-old) and aged (12–22 months-old) male and female mice. Atrophy was not present in the aged mice. Compared with adult mice, EDL muscles of aged mice had significantly lower specific force, longer tetanus relaxation times, and lower fatiguability. In the properties of absolute force and muscle relaxation times, females were affected by ageing to a greater extent than males. Additionally, EDL muscles from a separate group of male mice were subjected to eccentric contractions of 15% strain, and larger force deficits were found in aged than in adult mice.

**Conclusion:**

Our findings provide further insight into the muscle atrophy, weakness and fatiguability experienced by the elderly. We have shown that even in the absence of muscle atrophy, there are definite alterations in the physiological properties of whole fast-twitch muscle from ageing mice, and for some of these properties the alterations are more pronounced in female mice than in male mice.

## Introduction

Ageing in humans is accompanied by diminished function of the musculoskeletal system [1]. Between the ages of 40 and 80 years, muscle mass declines by 30 to 50% in both men and women [2]. This is accompanied by a decrease in muscle strength [3]–[4], an increase in fatiguability [5], and an increase in the susceptibility to contraction-induced damage [2]. The resulting impairments in mobility can lead to an increased risk of falls and declining quality of life [6]–[7].

The mechanisms underlying age-related deterioration in motor performance are complex and involve neural factors (both central and peripheral) and muscle-related factors [8]. One advantage of studying the effects of ageing in animals, rather than humans, is the ability to more easily differentiate between neural and muscle-related factors [9]. *In vitro* preparations of whole isolated muscle remove the influence of neural factors, so that age-related changes can be attributed to changes within the muscle tissue itself. They also allow more accurate quantitation of parameters such as muscle mass and cross-sectional area than can be achieved in human studies.

As in humans, studies on rodents indicate a significant degree of muscle atrophy with advancing age. A decline in the mass of hindlimb muscles from aged rodents has been observed by various investigators [10]–[15]. However, the onset of atrophy appears to be at a rather advanced age. Brown & Hasser [11] observed that significant falls in the mass of rat hindlimb muscles occurred only after 28 months of age. In mice, significant decreases in muscle mass have been reported at ages of 26–27 months [10] to 34–37 months [12]. Assuming an average lifespan of around 36 months in rats [16] and 30 months in mice [17], these results suggest that atrophy only becomes significant in the final 20% or so of the rodent lifespan.

Along with muscle atrophy, muscles of rodents undergo various changes in contractile characteristics with advancing age. There are age-related declines in total force, force per unit cross-sectional area, or both [10]–[16], [18]–[19]. This appears to be a result of both the loss of muscle mass and a loss in the intrinsic force-generating capacity of the muscle. Ageing is also associated with a slowing of muscle contraction and relaxation, with longer twitch contraction times [11], [18] and longer twitch half-relaxation times [10]–[11]. This could be partly due to the shift in fibre type distribution from fast (type II) to slow (type I) fibres that has been reported in the muscles of ageing rodents [11], [20]–[22]. Ageing muscle is also more susceptible to contraction-induced injury. When ageing muscles are subjected to eccentric contractions (contractions with stretch), they experience greater damage than muscles from younger animals [14]–[15], [23].

It is not entirely clear whether such changes in the contractile properties of ageing muscle are a consequence of the muscle atrophy, or whether they in fact occur before there is a significant loss of skeletal muscle tissue. Our aim in this present study, therefore, is to investigate the contractile function of whole isolated muscles from aged mice before the onset of significant muscle atrophy. We examine the following muscle parameters: mass, cross-sectional area, force, twitch and tetanus relaxation, force-frequency relationships, fatiguability, force loss following eccentric contractions, and stiffness. In our study, the aged mice are 12–22 months old. Significant muscle atrophy most likely occurs at a later age than this, as described above. By comparing the contractile properties of our aged mice with those of adult mice (2–6 months old), we aim to see whether there are any age-related changes in these contractile properties and whether these changes occur before muscle atrophy becomes established.

The muscle chosen for the study was the fast-twitch extensor digitorum longus (EDL) muscle from the hindlimb. This muscle was chosen because, as described above, there appears to be a shift towards slower-twitch characteristics in ageing muscle. As the EDL muscle is composed almost entirely of fast-twitch (type II) fibres [24], one would expect that shifts towards slower-twitch properties would be more apparent in this muscle. Additionally, the EDL muscle is more prone to eccentric damage than muscles containing a high proportion of slow-twitch fibres, such as the soleus [25], possibly because the larger diameter of fast-twitch fibres makes them more vulnerable to damage during contractile activity [26]. Hence by choosing the EDL it may be easier to detect differences in eccentric damage with age.

In this study we will examine muscles from both male and female mice. Previous studies on the contractile properties of ageing rodent whole muscle have examined only males [10]–[14], [16], [18] or females [15], [19]. To our knowledge, our present study is the first to examine muscles of both male and female mice under identical experimental conditions. It is important to compare males and females as there is evidence that ageing affects the muscles of male and female rodents differently, possibly because of hormonal factors [19].

## Methods

### Ethics statement

Use of animals was approved by the Animal Care and Ethics Committee of the University of New South Wales (Ethics approval number ACEC 08/119A). All animals were anaesthetised with halothane and sacrificed by cervical dislocation.

### Animals used

To assess mass, cross-sectional area, force, twitch and tetanus relaxation, force-frequency relationships, and fatiguability, we used a set of male and female 129/ReJ mice divided into two age groups: adult (2–6 months of age) and aged (20–22 months of age). To assess eccentric contractions and muscle stiffness, we used a separate group of male C57BL/10 mice divided into two age groups: adult (2 months of age) and aged (12 months of age).

### Muscle preparation

The extensor digitorum longus (EDL) muscle was dissected from the hindlimb, then tied by its tendons to a force transducer (World Precision Instruments, Fort 10; sensitivity 2200 µV/V/g, resonant frequency 300 Hz) at one end and a fixed metal hook at the other, using silk suture (Deknatel 6.0). It was placed in a bath continuously superfused with Krebs solution, with composition (mM): 4.75 KCl, 118 NaCl, 1.18 KH_2_PO_4_, 1.18 MgSO_4_, 24.8 NaHCO_3_, 2.5 CaCl_2_ and 10 glucose, with 0.1% fetal calf serum and continuously bubbled with 95% O_2_–5% CO_2_ to maintain pH at 7.4. The muscle was stimulated by delivering a supramaximal current between two parallel platinum electrodes, using an electrical stimulator (A-M Systems). At the start of the experiment, the muscle was set to the optimum length *L*_0_ that produced maximum twitch force. All experiments were conducted at room temperature (∼22°C to 24°C).

### Twitch parameters

The muscle was stimulated with a supramaximal pulse of 1 ms duration and the resulting twitch recorded. The twitch data was smoothed by averaging the raw data over 2.5 ms intervals, and from the resulting smoothed data the time-to-peak (time taken to reach peak twitch force) and half-relaxation time (time taken to relax to half of peak twitch force) were obtained.

### Force-frequency curve

A force-frequency curve was obtained by delivering 500 ms stimuli of different frequencies (2, 15, 25, 37.5, 50, 75, 100, 125 and 150 Hz), and measuring the force produced at each frequency of stimulation. A 30 second rest was allowed between each frequency. A curve relating the muscle force *P* to the stimulation frequency *f* was fitted to these data. The curve had the following equation [27]:
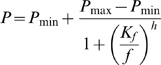
The values of *r* ^2^ for the fitting procedure were never lower than 99.3%. From the fitted parameters of the curve, the following contractile properties were obtained: maximum force (*P*_max_), half-frequency (*K_f_*), Hill coefficient (*h*) and twitch-to-tetanus ratio (*P*_min_/*P*_max_).

### Tetanus relaxation

Tetanus relaxation consists of a slow linear phase followed by a fast exponential phase. The linear phase is the easier to interpret as relaxation during this phase is homogeneous along the fibre, whereas in the exponential phase some parts of the fibre are lengthening and others are shortening [28]. In our measurements, the start of the linear phase was defined to be the point at which force began to fall following cessation of stimulation. Linear regression was then performed between this point and all subsequent points. The point at which the linear regression began to yield an *r* ^2^ of less than 98.5% was defined to be the end of the linear phase. We used the duration of this linear phase and the rate of force decline over this phase as measures of the rate of relaxation following a tetanus. The tetanus analysed was the 125-Hz tetanus from the force-frequency curve. Following the fatigue protocol (described in “Fatigue” below), a second force-frequency curve was obtained and the 125-Hz tetanus from this curve was also analysed.

### Fatigue

Muscles were given a one-second, 100-Hz tetanus every 2 seconds over a period of 30 seconds. The decline in 100-Hz force was tracked over this time as an indication of muscle fatiguability.

### Mass and cross-sectional area

At the end of the experiment, the muscle was removed from the bath. The tendons were trimmed and the muscle was lightly blotted on filter paper and then weighed to obtain wet muscle mass. An estimate of the cross-sectional area was obtained by dividing the muscle's wet mass by the product of its optimum length and the density of mammalian muscle (1.06 mg/mm^3^) [29].

### Eccentric contractions

In a separate group of mice, EDL muscles were subjected to an eccentric contraction protocol. Muscles were prepared as described in “Muscle preparation” above. The optimum length *L*_0_ was 11.7±0.3 mm in muscles from adult mice and 13.0±0.0 mm in muscles from aged mice (*P*  =  0.0189). Prior to the eccentric contractions, a force-frequency curve was generated as described in “Force-frequency curve” above. The eccentric contraction protocol was then performed. At time  =  0 ms, the muscle was stimulated by supramaximal pulses of 1 ms duration and 100 Hz frequency. At time  =  750 ms, after it had attained its maximum isometric force, the muscle was stretched at a speed of 1 mm/s until it was 15% longer than its optimum length, held at this length for 2 seconds, then returned at the same speed to its original position. The electrical stimulus was stopped at time  =  5000 ms. This eccentric contraction was performed 3 times, at intervals of 5 minutes. Twenty minutes after the final eccentric contraction, the setting of the optimum length *L*_0_ was repeated. In all muscles, *L*_0_ was the same before and after eccentric contractions. A second force-frequency curve was then obtained, and the force deficit was calculated as the percentage fall in *P*_max_ between the first and the second force-frequency curves.

### Statistical analyses

Data are presented as Mean ± S.E.M.. All statistical comparisons between adult and aged mice were made using two-tailed *t*-tests, with the exception of the eccentric contraction and muscle stiffness data, where Mann-Whitney tests were used due to smaller sample sizes. Statistical significance was defined as *P*<0.05. All statistical tests and curve fitting were performed using a standard statistical software package (GraphPad Prism Version 5 for Windows, GraphPad Software, San Diego California USA).

## Results

### Mass and cross-sectional area

**Figure 1:**
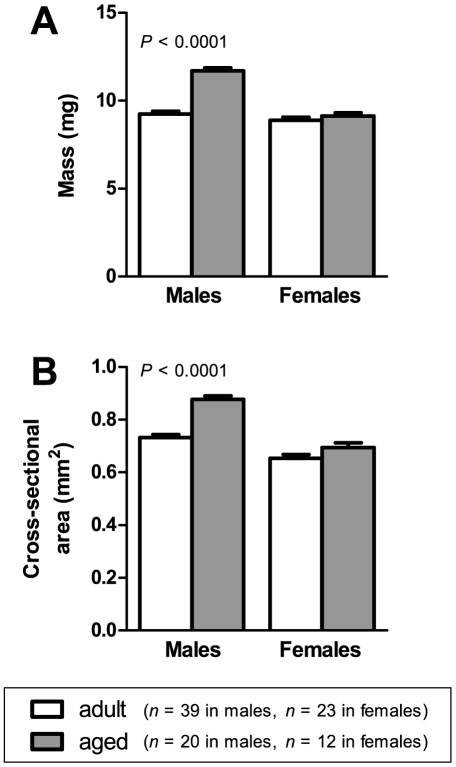
Mass and cross-sectional area. In males, EDL muscles of aged animals had higher mass (A) and cross-sectional area (B) than muscles from adult animals. In females, there were no significant differences between adult and aged animals.

**Figure 1** shows the mass **(A)** and cross-sectional area **(B)** of EDL muscles from adult and aged male and female mice. In males, the muscles of aged mice had 27% greater mass (*P*<0.0001) and 21% larger cross-sectional area (*P*<0.0001) than muscles from adult mice. In females however, mass and cross-sectional area were not significantly different between adult and aged mice.

### Maximum forces

**Figure 2:**
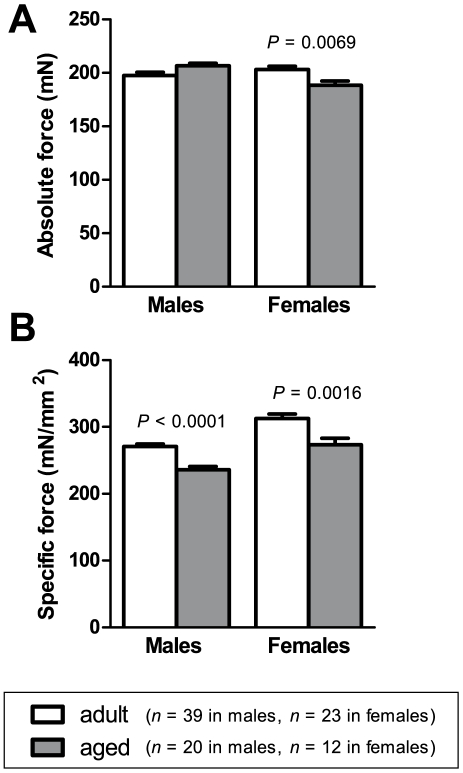
Maximum forces. In females, EDL muscles of aged animals had lower absolute force (A) than muscles from adult animals. Specific force (B) was lower in aged compared with adult animals, in both males and females.

**Figure 2** shows the maximum absolute force **(A)** and the maximum specific force **(B)** generated by the EDL muscles of adult and aged male and female mice. Absolute forces in males were no different between adult and aged mice. In females, muscles from aged mice showed a 7.2% lower absolute force compared with adult mice (*P*  =  0.0069). Muscles from aged animals generated significantly lower specific force than muscles from adult animals, in both males and females. The difference was 13% in males (*P*<0.0001) and 13% in females (*P*  =  0.0016).

### Twitch parameters

**Figure 3:**
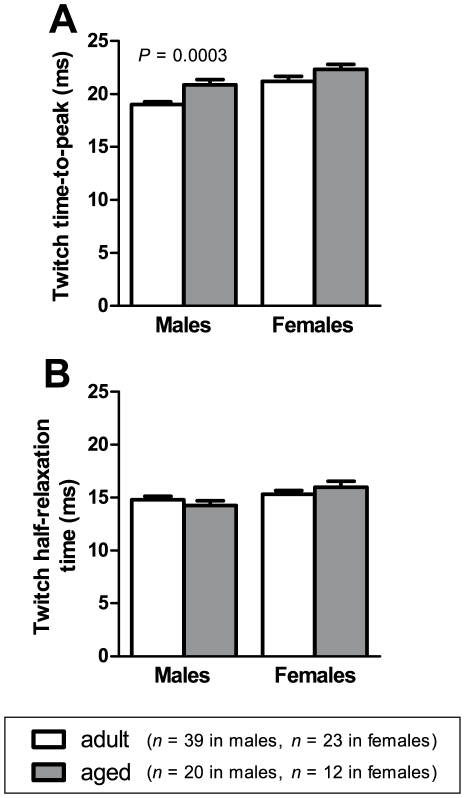
Twitch parameters. Time-to-peak (A) was longer in EDL muscles of aged male mice than in adult male mice. There were no differences in twitch half-relaxation time (B) between aged and adult mice, in either males or females.

**Figure 3** shows twitch time-to-peak **(A)** and half-relaxation time **(B)** for EDL muscles from adult and aged male and female mice. In males, time-to-peak was 1.9 ms longer in muscles from aged animals compared with muscles from adult animals (*P*  =  0.0003). No differences in half-relaxation time were observed between adult and aged animals, for either males or females.

### Tetanus relaxation

**Figure 4:**
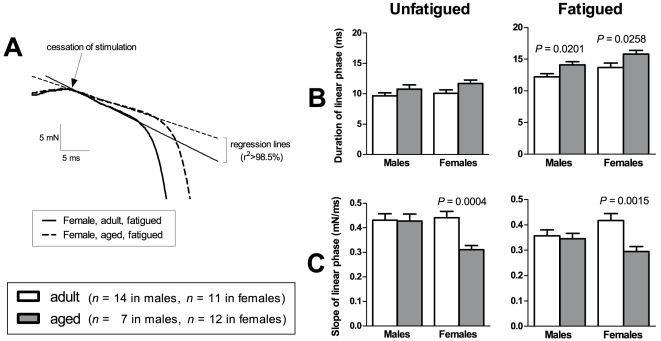
Tetanus relaxation. (A) shows force recordings of the tetanus for two EDL muscles in our sample. It shows the final stages of the period of stimulation, and the initial stages of relaxation. It can be seen that force declines linearly in the initial stages of relaxation. In the muscle that relaxes more slowly (dashed line), this linear phase has a longer duration and a reduced steepness of slope compared with the faster-relaxing muscle (full line). We examined this linear phase both before and after subjecting the muscles to a fatiguing stimulation protocol. The duration of the linear phase is shown in (B) and the steepness of the slope of this linear phase is shown in (C).

**Figure 4** shows our analysis of relaxation following tetanic stimulation in the EDL muscles of adult and aged male and female mice. Relaxation following tetanic stimulation generally occurs in two phases – an initial phase where force declines linearly, followed by a faster phase where force declines exponentially [28]. The intial linear phase is easier to interpret in terms of muscle relaxation [28] and hence this is the phase we have chosen to analyse. **(A)** is a recording of tetanus relaxation in two EDL muscles from our sample, showing muscle force in the final stages of stimulation and the initial stages of relaxation. Regression lines have been drawn to indicate the linear phase of relaxation. We measured the duration and slope (rate of force decline) of this linear phase, both before and after subjecting our sample of muscles to a fatiguing stimulation protocol (see *Methods* for fatigue protocol). The results for duration are shown in **(B)** and the results for rate of force decline are shown in **(C)**.

In unfatigued muscles, there were no significant differences between adult and aged animals in the duration of the linear phase. However, in females, the rate of force decline was significantly lower in aged than in adult mice (*P*  =  0.0004). In fatigued muscles, the duration of the linear phase was significantly longer in aged than in adult animals, in both males (*P*  =  0.0201) and females (*P*  =  0.0258). Also, in fatigued muscles of females, the rate of force decline was significantly lower in aged than in adult mice (*P*  =  0.0015).

### Force-frequency curve

**Figure 5:**
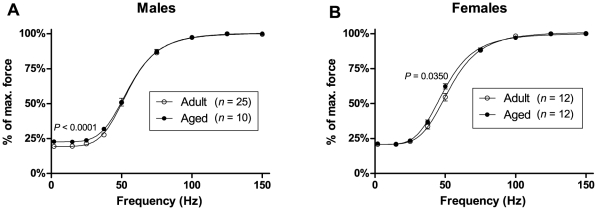
Force-frequency curves. Force-frequency curves for EDL muscles from adult and aged animals are shown in (A) for males and (B) for females. In males, the force-frequency curve in aged animals is shifted upwards at low frequencies, reflecting a significantly higher twitch-to-tetanus ratio than in adult animals. In females, the force-frequency curve for aged mice is shifted leftwards, reflecting a significantly lower half-frequency than in adult animals.

**Figure 5** shows the force-frequency curves for EDL muscles from adult and aged mice, for males **(A)** and females **(B)**. In males, the force-frequency curve for aged mice is shifted upwards at low frequencies compared with the curve for adult mice, reflecting a higher twitch-to-tetanus ratio in aged mice (22.5±0.7% for aged, 19.3±0.4% for adult, *P*<0.0001). In females, the force-frequency curve for aged mice is shifted leftwards compared with the curve for adult mice, reflecting a lower half-frequency in aged mice (49.7±0.9 Hz for aged, 53.6±1.4 Hz for adult, *P*  =  0.0350).

### Fatigue

**Figure 6:**
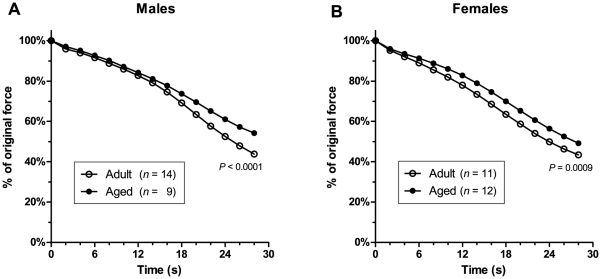
Fatigue. The time course of force decline during 30 seconds of fatiguing stimulation is shown in (A) for males and (B) for females. It can be seen that in both males and females, EDL muscles from aged animals fatigued less rapidly than muscles from adult animals. At the end of the 30-second fatigue protocol, muscles from aged animals were able to generate a significantly higher percentage of their pre-fatigue force than muscles from adult animals. (Error bars are within thickness of symbols.)

**Figure 6** shows the decline in 100-Hz force in EDL muscles during a stimulation protocol consisting of a 1-second, 100-Hz tetanus given every 2 seconds over a period of 30 seconds. In both males **(A)** and females **(B)**, force declines less rapidly in muscles from aged animals than in muscles from adult animals, indicating a greater fatigue resistance in muscles from aged animals. At the end of the 30-second protocol, muscles of aged animals were able to generate a significantly higher percentage of their pre-fatigue force than muscles of adult animals (males – 54.2±1.3% for aged, 43.8±1.1% for adult, *P*<0.0001; females – 49.2±1.2% for aged, 43.5±0.7% for adult, *P*  =  0.0009).

### Eccentric contractions and muscle stiffness

**Figure 7:**
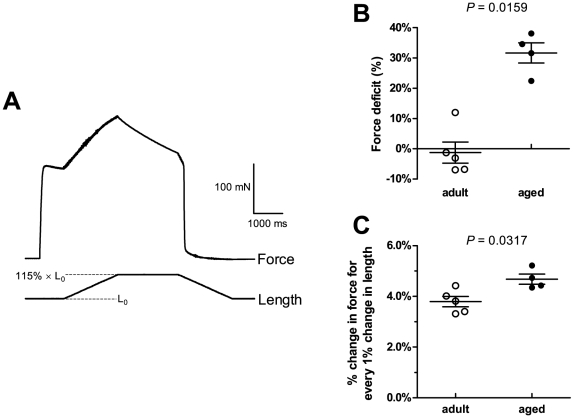
Eccentric contractions and stiffness. In a separate group of mice, EDL muscles were subjected to a mild eccentric contraction protocol. (A) is an example of an eccentric contraction, showing the change in force and length as the muscle is stretched by 15% of its optimal length L_0_, then returned to resting length. 3 such eccentric contractions were performed for each muscle. (B) shows the force deficits following these eccentric contractions. Force deficits in aged mice were significantly higher than in adult mice. As an indicator of muscle stiffness, we also measured the percentage change in force for every 1% change in length during the stretch phase of the eccentric contraction. The results are shown in (C). The change in force was significantly higher in aged than in adult mice, indicative of greater stiffness in the muscles of aged mice.

In a separate group of mice, we examined the susceptibility of EDL muscles to damage from a mild eccentric contraction protocol of 15% strain. An example of an eccentric contraction is shown in panel **(A)** of **Figure 7**. The muscle is first stimulated to contract isometrically at its optimal length. While stimulation continues, it is then stretched by 15% of its optimum length, and held in this stretched position. Stimulation is then stopped and the muscle returned to its original length. 3 such eccentric contractions were performed for each muscle.

As an indicator of muscle damage, we measured the force deficit, which is the percentage drop in force following the eccentric contractions. The results are shown in **(B)**. Muscles of adult animals experienced virtually no drop in force following the eccentric contractions. However, aged animals lost 32±3.4% of their original force, suggesting a greater susceptibility to eccentric contraction-induced damage in aged compared with adult animals (*P*  =  0.0159).

As an indicator of muscle stiffness, we measured the change in force as the muscle was lengthened during the first eccentric contraction. Force was expressed as a percentage of the isometric force before stretching, and length was expressed as a percentage of optimum length. The results are shown in **(C)**. For every 1% increase in length, there was a 3.8±0.2% increase in force in muscles from adult animals, and a 4.7±0.2% increase in force in muscles from aged animals. Hence muscles from aged animals had greater stiffness than muscles from adult animals (*P*  =  0.0317).

### Analysis of ramp phase of eccentric contractions

The observed difference in whole muscle stiffness between adult and aged animals could arise from differences in contractile proteins, or from differences in non-contractile components such as titin or the extracellular matrix. To investigate whether the contractile proteins contribute to the observed age-related change in muscle stiffness, we analysed the ramp phase of the first eccentric contraction in each muscle. This is the phase during which the muscle is being stretched. The way in which the force changes during this phase is a reflection of the crossbridge interactions between the contractile proteins actin and myosin [30]–[32].

**Figure 8:**
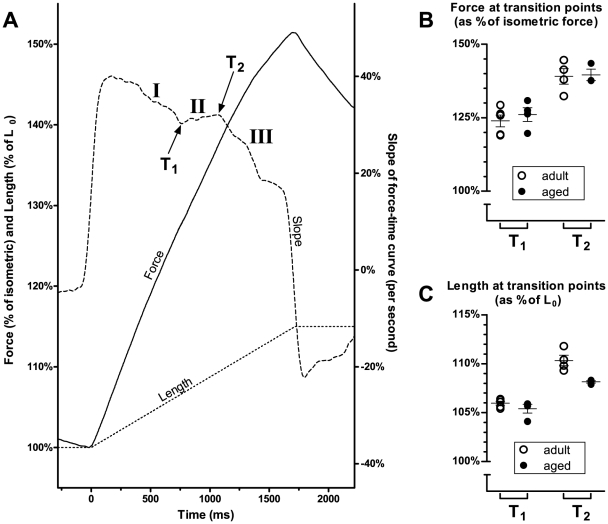
Analysis of ramp phase of eccentric contractions. The diagram in (A) shows the change in force (full line) and length (dotted line) as the muscle is stretched during an eccentric contraction. The dashed line is the slope (first derivative) of the force-time curve. The plot of slope exhibits three distinct phases, and each phase is separated by a transition point (T_1_ and T_2_). For each muscle, we measured the force and length at T_1_ and T_2_. The results are shown in the graphs on the right. There were no significant differences between adult and aged animals in either the force (B) or length (C) at which the transition points occurred.

Our analysis is shown in **Figure 8**. Panel **(A)** shows the ramp phase of the first eccentric contraction in one muscle from our sample. It shows changes in force (full line) and length (dotted line) as the muscle is being stretched. The dashed line is the slope (first derivative) of the force-time curve. The slope can be seen to change over three stages: I, in which the slope declines; II, in which the slope remains constant and in some cases increases; and III, in which the slope declines again. Between each stage is a transition point (T_1_ and T_2_).

The transition points identified here are qualitatively similar to those described by other authors. An abrupt reduction in the slope of the force-time curve, likely to correspond to T_2_ in Figure 8(A), has been observed in rat flexor hallucis brevis (FHB) [31], frog sartorius [30] and cat soleus [32]. It is thought that this transition represents the point at which strained crossbridges are forcibly detached. Up to this point, the tension rise is due to the crossbridges being increasingly strained as the muscle is stretched; past this point, the tension rise is due to non-crossbridge components within the muscle [30], [33]. Flitney & Hurst [30] and Pinniger *et al.* [31] also identify a point of inflection in the force-time curve prior to T_2_ being reached. It is likely that this corresponds to T_1_ in Figure 8(A), and it is thought to arise from myosin heads undergoing a reverse power stroke as the muscle is stretched [33].

Hence differences in transition points between muscles could indicate that there are differences in the nature of the crossbridge interactions, such as the ability of the crossbridges to resist stretching. We therefore determined T_1_ and T_2_ in each muscle, using the approach outlined in Figure 8(A), and recorded: (i) the muscle length at which T_1_ and T_2_ occurred (as a percentage of optimum length); and (ii) the muscle force at which T_1_ and T_2_ occurred (as a percentage of isometric force).

The results are shown in panel **(B)** for muscle length and panel **(C)** for muscle force. No significant differences were found between the muscles of adult and aged animals in either the length or the force at which their transition points occurred. This suggests that crossbridge interactions between the contractile proteins was not a contributor to the observed difference in whole muscle stiffness between adult and aged mice.

## Discussion

In humans, ageing causes skeletal muscles to become atrophied, weak, fatiguable, and easily damaged by eccentric contractions [2]. In this study we have examined the contractile properties of isolated fast-twitch EDL muscles to see whether such changes occur in the muscles of aged male and female mice.

### Muscle atrophy

Muscle atrophy was not present in our aged mice. In females, muscle mass and cross-sectional area were no lower in aged mice than in adult mice, while in males these parameters were actually higher in aged mice than in adult mice (Figure 1). Our aged mice were 19–22 months old. Hence it appears that at this age, muscle atrophy has not yet become established. Indeed, it has been reported that significant atrophy in rat hindlimb muscles does not start until 28 months of age [11] (about the final 20% of the average lifespan). Studies that have observed a lower EDL mass in aged mice than in younger (3–6 month-old) mice have used mice that were older than those in our study (26–27 months old in Brooks & Faulkner [10]; 34–36 months old in Pagala *et al.* [12]). The age of the mice in our study was thus appropriate for investigating whether the contractile properties of EDL muscle change before there is significant muscle atrophy.

Even though the muscles of our aged mice were not atrophied compared with the muscles of adult mice, we still found age-related changes in EDL contractile characteristics. We found a fall in force-generating capacity, a shift towards slower-twitch characteristics, and a reduction in fatiguability. These changes occurred in the absence of any reduction in muscle mass or cross-sectional area.

### Fall in force-generating capacity

There was a decline in the force-generating capacity of EDL muscle with age. Absolute force decreased with age in the muscles of adult females (Figure 2A), while specific force (force per unit cross-sectional area) decreased with age in both males and females (Figure 2B). These results suggest that muscle weakness in the elderly may not be due solely to atrophy, but there is an age-related decline in the intrinsic force-generating capacity of the muscle itself.

The decline in muscle specific force with age is a highly consistent finding among studies on rodents [10]–[11], [13]–[16]. The loss of force per unit cross-sectional area may be partly due to an increase in the amount of extracellular non-contractile tissue in skeletal muscles with age [13]. However, the specific force of individual fibres also declines with age [9], [34]–[35], suggesting that the loss of muscle force is also due to factors intrinsic to the muscle fibres themselves. Possible factors include: structural alterations to myosin [36], changes in actin-myosin crossbridge kinetics [35], and impairments in excitation-contraction coupling [34], [37]–[38].

### Shift towards slower-twitch characteristics

We found changes in EDL muscle that suggest an age-related shift towards slower-twitch characteristics. In males, the time-to-peak of the twitch was longer in aged mice compared to adult mice (Figure 3A). Relaxation following a tetanus was also slower in aged mice than in adult mice. Our analysis of the linear phase of tetanus relaxation (Figure 4) showed that this linear phase lasted longer in aged mice than in adult mice (Figure 4B), and the rate of force decline during this phase was lower in aged compared to adult mice (Figure 4C), although this difference in decline rate was found in females only. The force-frequency curve for EDL muscles of females (Figure 5B) was also shifted to the left in aged mice, which is a change characteristic of slower-twitch muscles. The force-frequency curves of slow-twitch muscles are shifted to the left of those for fast-twitch muscles since twitches in slow-twitch muscles summate at lower frequencies [18]. Our twitch, tetanus and force-frequency data thus present an overall picture of EDL muscles becoming more slow-twitch in nature with increasing age.

Other studies have also reported a shift towards slower-twitch characteristics with advancing age [11], [18]–[19]. One possible reason for the development of slower-twitch characteristics is the reduction in the proportion of fast fibres, especially type IIb fibres, and accompanying increase in the proportion of slow fibres that has been reported to occur in ageing muscle [22]. This appears to be due in part to the denervation of fast fibres and their reinnervation by axonal sprouting from slow fibres [20]–[21]. Additionally, the slower relaxation of the aged muscles in our study could also be due to a reduced rate of Ca^2+^ uptake by the sarcoplasmic reticulum (SR) [39], and indeed SR Ca^2+^-ATPase activity has recently been shown to be reduced in aged rat diaphragm muscle [40]. Mechanically skinned fibres from aged EDL mice also show impaired excitation-contraction coupling and impaired Ca^2+^ release from the SR [41], which would lead to a slower twitch response.

It is interesting that the age-related slowing of muscle contractile properties in our study was more pronounced in the female mice than in males. The age-related reduction in tetanus decline rate (Figure 4C) and left-shifting of the force-frequency curve (Figure 5B) were found only in females. The more pronounced effects of ageing on our female mice compared to our male mice may be due to falling levels of ovarian hormones. Ovarian failure occurs at around 11 to 16 months of age in mice [42], and so the aged female mice in our study were post-ovarian failure. Various studies (reviewed in Lowe *et al.* [42]) have demonstrated that falling ovarian hormone levels have a detrimental effect on muscle strength in rodents, through mechanisms such as reducing the fraction of strong-binding myosin during contraction [43]–[44], and indeed in our study we observed a loss of absolute muscle force in aged females but not in aged males (Figure 2A). It is possible that, as well as affecting muscle strength, declining hormone levels also have an impact on the twitch and tetanus characteristics of a muscle.

### Reduced fatiguability

In humans, ageing usually causes people to feel fatigued more quickly when performing everyday tasks [2], [5]. In rodents also, ageing results in poorer endurance ability in activities such as treadmill running and swimming [12]. It is interesting, therefore, that in our study we found a reduced fatiguability of EDL muscles with age. In both males and females, the rate of force decline during a 30-second fatigue protocol was significantly less in muscles of aged mice than in muscles of adult mice (Figure 6). In reconciling these results with the experience of increased fatiguability in aged humans and aged mice, a distinction must be made between central fatigue, which is due to decreased activation from the central nervous system (CNS), and peripheral fatigue, which is due to factors within the skeletal muscles themselves [45]. There is evidence that much of the increased fatiguability in elderly humans [46]–[48] and aged mice [12] is actually due to an increase in central fatiguability with age. This may involve reduced supraspinal drive [49] due to degradation and reduced excitability of cortical motoneurons [50]–[51].

As our isolated muscle procedure removes any CNS effects, our results clearly demonstrate that there is a reduction in the peripheral fatiguability of the EDL muscle with age. These results are in accord with those of Brown & Hasser [11] who also found reduced fatiguability in aged EDL rodent muscle, and with human studies which show that peripheral fatigue may develop more slowly in the elderly [46]–[47].

The reduced peripheral fatiguability of ageing muscle may be due to the loss of fast-twitch muscle fibres and their replacement by slow-twitch muscle fibres which are more resistant to fatigue [20]–[21]. Gonzalez & Delbono [52] found that the fatiguability of individual fibres from mouse EDL muscle does not change significantly with age, suggesting that the reduced fatiguability of ageing whole muscle may be due to changes in fibre type composition, rather than changes within the muscle fibres themselves. Likewise, Brown & Hasser [11] found a reduced fatiguability of rat EDL muscle with age and an accompanying reduction in the proportion of the fibre area occupied by type IIb fibres.

When whole isolated muscles are subjected to intense, repeated stimulation, as they were in this study, K^+^ can accumulate within the lumen of the t-tubules, leading to a rise in extracellular [K^+^] and a reduction in membrane excitability [53]. Hence it is possible that some of the force decline observed during our fatigue protocol was caused by a loss of membrane excitability. Thus the smaller force decline in our aged muscles could also reflect an age-related increase in the densities of the Na^+^/K^+^ ATPase in the t-tubule membrane. However, further studies would be needed to determine if there are in fact any differences in t-tubular Na^+^/K^+^ ATPase densities with age.

### Eccentric contractions

In humans, ageing is associated with increased muscle damage following eccentric contractions [2]. Incomplete recovery from contraction-induced muscle injury is likely to be one contributor to the loss of muscle mass and force with ageing [14]–[15]. In a separate group of mice, we compared the loss of force in EDL muscles of adult and aged mice immediately following a mild eccentric contraction protocol of 15% strain (Figure 7). While this protocol did not damage muscles of adult mice, it caused a force deficit of about 30% in aged mice (Figure 7B). This indicates an increased susceptibility of ageing muscle to eccentric contraction-induced injury. Moreover, the aged mice in the eccentric contraction experiments were 12 months old. This is well before the onset of muscle atrophy (as discussed above), so the increased susceptibility to contraction-induced damage occurs well before there is a significant loss of muscle mass. Our results are consistent with those of Brooks & Faulkner [23], who found that EDL muscles from aged mice experienced higher force deficits than muscles from younger mice for any given level of work input. Single permeabilised fibres from EDL muscles of aged rats also show higher force deficits than fibres from younger rats following eccentric contractions [23], [54].

To investigate whether differences in muscle stiffness might be contributing to ageing muscle's increased susceptibility to contraction-induced damage, we estimated muscle stiffness by the change in force occurring for a given length change during the stretch phase of the eccentric contractions. We found that, for a given change in length, muscles from aged mice experienced a larger change in force than muscles from adult mice (Figure 7C), indicating that ageing is associated with an increase in active muscle stiffness. This may contribute to the age-related increase in muscle damage as a stiffer muscle is less compliant and less capable of absorbing mechanical strains during eccentric contractions.

All muscles were stretched at a velocity of 1 mm/s. Expressed in terms of fibre lengths per second (*L_f_*/s), this equates to a velocity of 0.20±0.01 *L_f_*/s in adult mice and 0.17±0.00 *L_f_*/s in aged mice, assuming a fibre length to muscle length ratio of 0.44 [55]. Because the change in force during an eccentric contraction is dependent on stretch velocity (in *L_f_*/s) [55], it might be argued that the different velocities of stretch in adult and aged mice may have confounded our estimate of muscle stiffness. However, a higher velocity (in *L_f_*/s) actually leads to a larger change in force [55], so if the muscles of aged mice had been stretched at the same velocity (in *L_f_*/s) as the muscles of adult mice, it is likely that we would have observed an even greater change in force in the aged muscles. Hence the difference in estimated muscle stiffness between adult and aged mice may be even greater than what we have reported.

The greater stiffness of whole muscle in our aged mice could arise from changes in actin-myosin crossbridge properties, or from changes in non-contractile components such as titin or the extracellular matrix. To investigate whether crossbridge interactions during eccentric contractions were any different between adult and aged mice, we analysed the stretch (ramp) phase of the eccentric contractions (Figure 8). We identified two transition points in the slope of the force-time relationship (Figure 8A). As explained in the *Results*, these transition points are likely to reflect the properties of actin-myosin crossbridges. There were no differences between the muscles of adult and aged mice in either the force or the length at which these transition points occurred (Figure 8B), indicating that, during eccentric contractions, actin-myosin crossbridge properties were similar in both adult and aged mice. Hence the increased stiffness of the aged EDL muscles in our study was probably due to an increased stiffness of non-contractile components such as titin or the extracellular matrix.

Of the two transition points, the second point (referred to here as T_2_) is the one more consistently observed in other studies [31]. In our muscles, T_2_ occurred when force had reached around 140% of isometric force, which is comparable to other studies [30]–[31]. However, the length at which T_2_ occurred in our muscles (about 110% of optimum length) was greater than the 101%–102% observed by Pinniger *et al.* [31] in rat FHB and Flitney & Hirst [30] in frog sartorius. We used a highly objective, quantitative method to determine the transition points from our force tracings (by taking the first derivative of the force-time curve), as opposed to the other studies where the transition points were determined by visual inspection, and by our method we did not observe any transition points at 101%–102% of optimum length. The discrepancy in this value between our study and other studies is most likely due to different compliances of the muscles tested and of the experimental setups.

### Conclusion

Our study has shown that even in the absence of muscle atrophy, there are definite alterations in the physiological properties of whole fast-twitch muscle from ageing mice. In these muscles, ageing is associated with a fall in force production per unit cross-sectional area and a shift towards slower-twitch properties, evidenced by a slowing of relaxation and reduced fatiguability. For absolute force and muscle relaxation, age-related changes appeared to affect female mice to a greater extent than male mice, possibly due to hormonal factors. Ageing was also associated with an increased susceptibility to muscle damage induced by eccentric contractions. These findings provide further insight into the muscle atrophy, weakness and fatiguability experienced by the elderly.
